# Long-term and pathological outcomes of low- and intermediate-risk prostate cancer after radical prostatectomy: implications for active surveillance

**DOI:** 10.1007/s00345-021-03717-2

**Published:** 2021-05-10

**Authors:** Valentin H. Meissner, Mira Woll, Donna P. Ankerst, Stefan Schiele, Jürgen E. Gschwend, Kathleen Herkommer

**Affiliations:** 1 Department of Urology, Klinikum rechts der Isar, School of Medicine, Technical University of Munich, Ismaninger Strasse 22, 81675 Munich, Germany; 2 Departments of Mathematics and Life Sciences, Technical University of Munich, Munich, Germany

**Keywords:** Active surveillance, Favorable intermediate risk, Oncological outcome, Radical prostatectomy, Prostate cancer

## Abstract

**Purpose:**

The safety of active surveillance (AS) in favorable intermediate-risk (FIR) prostate cancer (PCa) remains uncertain. To provide guidance on clinical decision-making, we examined long-term and pathological outcomes of low-risk and intermediate-risk PCa patients after radical prostatectomy (RP).

**Methods:**

The study involved 5693 patients diagnosed between 1994 and 2019 with low-risk, FIR, and unfavorable intermediate-risk (UIR) PCa (stratification according to the AUA guidelines) who underwent RP. Pathological outcomes were compared, and Kaplan–Meier analysis determined biochemical recurrence-free survival (BRFS) and cancer-specific survival (CSS) at 5, 10, 15, and 20 years. Multiple Cox regression was used to simultaneously control for relevant confounders.

**Results:**

Those at FIR had higher rates of upgrading and upstaging (12.8% vs. 7.2%, *p* < 0.001; 19.8% vs. 12.0%, *p* < 0.001) as well as pathological tumor and node stage (≥ pT3a: 18.8% vs. 11.6%, *p* < 0.001; pN1: 2.7% vs. 0.8%, *p* > 0.001) compared to patients at low risk. The 20-year BRFS was 69%, 65%, and 44% and the 20-year CSS was 98%, 95%, and 89% in low-risk, FIR, and UIR patients. On multiple Cox regression, FIR was not associated with a worse BRFS (HR 1.07, CI 0.87–1.32), UIR was associated with a worse BRFS (HR 1.49, CI 1.20–1.85).

**Conclusion:**

Patients at FIR had only slightly worse pathological and long-term outcomes compared to patients at low risk, whereas the difference compared to patients at UIR was large. This emphasizes AS in these patients as a possible treatment strategy in well-counseled patients.

**Supplementary Information:**

The online version contains supplementary material available at 10.1007/s00345-021-03717-2.

## Introduction

Active surveillance (AS) has become a widely accepted standard of care in low-risk prostate cancer (PCa) to reduce overtreatment and associated morbidity. AS offers the benefit of preservation of quality of life while retaining the assurance of definitive treatment if required. Cancer-specific survival (CSS) rates of AS in low-risk PCa have been reported to be consistent with those of radiation therapy or surgery [1, 2]. The use of AS in intermediate-risk PCa patients is steadily increasing [3]. Recently, various guidelines, including those of American Urological Association (AUA) and National Comprehensive Cancer Network (NCCN), have designated AS as an acceptable management strategy in men with favorable intermediate-risk (FIR) PCa [4–6]. Furthermore, multiparametric magnetic resonance imaging (mpMRI) showed improvements in risk stratification of men on AS and was recommended for enhancing enrollment and monitoring decisions [7, 8]. However, data on the safety of AS in these patients are inconsistent and limited relative to data supporting the safety of AS in men at low risk [9, 10]. A recent study reported on the safety of AS in the short term for selected and closely monitored men with Grade Group (GG) 2 PCa [11]. In contrast, a prospective cohort study reported on the feasibility of AS in FIR PCa patients with biopsy GG 1 and PSA greater than 10 ng/ml as a safe treatment strategy; the presence of biopsy GG 2, however, increased the risk of metastatic disease [9]. A further study reported higher rates of adverse pathological outcomes and shorter times to biochemical recurrence in FIR PCa patients compared to patients at low risk after radical prostatectomy (RP) [12]. Another large comparative cohort study of men treated with RP reported additionally worse overall survival in patients classified at FIR compared to low risk [13]. Results of these and previous studies regarding metastasis and survival outcomes did not comprehensively control for relevant confounders and were often limited by either the low number of patients included or the short duration of follow-up. Additionally, information on risk factors, such as ethnicity or a positive family history, were lacking.

To address this void and provide guidance on selecting FIR PCa patients for AS, this study examined long-term outcomes up to 20 years, comprising biochemical recurrence-free survival (BRFS), CSS and adverse surgical pathology, for low-risk and FIR PCa patients after RP, controlling for relevant confounders, including a detailed family history of cancer.

## Patients and methods

### Database and study population

Analyses were based on the nationwide multicenter German Familial Prostate Cancer prospective study, which has surveyed newly diagnosed patients with PCa independent of family history since 1994 [14, 15]. Informed consent was obtained from each patient. The study was approved by the ethical review committee of the Technical University of Munich. For the current analysis, we retrospectively identified patients diagnosed between 1994 and 2019 with low-risk, FIR or unfavorable intermediate-risk (UIR) histologically confirmed PCa treated with RP. Patients with neoadjuvant hormone therapy or other first-line therapies were excluded. As per AUA guidelines [5], low-risk PCa was defined as clinical T1c-T2a, biopsy GG 1, and PSA < 10 ng/ml, and FIR was defined as clinical T1c–T2a, biopsy GG 1, PSA 10–20 ng/ml or clinical T1c–T2a, biopsy GG 2, PSA < 10 ng/ml. An UIR group was also identified for comparison purposes as clinical T2b–T2c, biopsy GG 2, PSA < 10 ng/ml, or clinical T1c–T2c, biopsy GG 2, PSA 10–20 ng/ml, or clinical T1c–T2c, biopsy GG 3, PSA < 20 ng/ml. Gleason score was assigned according to the ISUP (International Society of Urological Pathology) Grade Group designations [16] following current practice.

Sociodemographic and clinical data included age at surgery, family history of PCa [hereditary according to the Johns Hopkins criteria [17], first-degree (1 first-degree relative with PCa), non (no first-degree relatives with PCa)], fatal family history of PCa, other cancer family history, secondary cancer, PSA at diagnosis, and digital rectal examination (DRE). Pathological and follow-up data included postoperative upgrading (defined as postoperative GG ≥ 3 tumor in RP) and upstaging (defined as pT3-pT4 or pN1 disease at RP), pathological tumor and node stage according to the TNM classification, surgical margin, pathological GG at RP, adjuvant radiotherapy, and adjuvant hormone therapy. Pathological staging was classified or reclassified for patients diagnosed before 2002 using the UICC TNM classification 2002. BRFS was defined as PSA ≤ 0.2 ng/ml and assessed by clinical reports. CSS was determined by clinical reports and verified by the course of the disease.

### Statistical analysis

Chi-square and Kruskal–Wallis tests were used to compare categorical and continuous variables between low-risk, FIR, and UIR PCa patients. Kaplan–Meier analysis was performed to determine BRFS and CSS rates at 5, 10, 15, and 20 years with 95% confidence intervals (CI) for the low, FIR and UIR groups. Potential prognostic factors for BRFS and CSS were examined using single Cox regression, with multiple Cox regression with backward elimination (selection level 5%) employed to simultaneously control for relevant confounders for the assessment of effect of risk group (low, FIR, UIR) on BRFS and CSS. Hazard ratios (HR) with 95% CIs and two-sided *p* values were calculated, with statistical significance set at the 0.05 level. All analyses were conducted using SAS 9.4.

## Results

Overall, 5693 patients were included for the analysis, comprising 45.8% (*n* = 2 607), 26.1% (*n* = 1 484), and 28.1% (*n* = 1602) low-risk, FIR, and UIR PCa patients, respectively (Table 1). Patients with FIR PCa were more likely to be postoperatively upgraded and upstaged compared to low-risk PCa patients (12.8% vs. 7.2%, *p* < 0.001 and 19.8% vs. 12.0%, *p* < 0.001). Overall stage and grade migration toward non-organ confined disease/lymph node invasion and higher GG increased over the study period (Supplementary Table 1). Those with UIR PCa were more likely to have adverse pathological features including pathological tumor stage (≥ pT3a: 37.5% vs. 18.8% and 11.6%, *p* < 0.001), pathological node stage (pN1: 8.8% vs. 2.7% and 0.8%, *p* > 0.001), and pathological GG at RP (GG ≥ 2: 89.9% vs. 62.3% and 29.4%, *p* < 0.001) compared to patients at FIR and low risk, respectively. Furthermore, adjuvant radiotherapy and hormone therapy were more often administered for UIR PCa patients compared to FIR PCa patients and those had more often adjuvant therapy compared to low-risk PCa patients (8.2% vs. 4.7% vs. 3.4%, *p* < 0.001 and 7.7% vs. 3.6% vs. 2.0%, *p* < 0.001) (Table 1).

**Table 1:** Comparison of demographic and clinical characteristics, and pathological outcomes of low-risk, FIR, and UIR PCa patients

Factor	Low-risk (*n* = 2607)	FIR (*n* = 1484)	UIR (*n* = 1602)	*p* value
Median age at surgery, y (IQR)	64.6 (8.4)	65.0 (9.0)	66.2 (8.7)	< 0.001
Family history of PCa, *n* (%)				< 0.001
Non	1931 (74.1)	1126 (75.9)	1230 (76.8)	
First-degree	465 (17.8)	283 (19.1)	296 (18.5)	
Hereditary	211 (8.1)	75 (5.0)	76 (4.7)	
Fatal family history of PCa, *n* (%)	104 (4.0)	53 (3.6)	38 (2.4)	0.020
Other cancer family history, *n* (%)	1323 (50.8)	704 (47.5)	758 (47.3)	0.041
Secondary urologic cancer, *n* (%)	83 (3.2)	56 (3.8)	46 (2.9)	0.357
Secondary non-urologic cancer, *n* (%)	264 (10.1)	123 (8.3)	124 (7.7)	0.018
Median PSA at diagnosis, ng/mL (IQR)	5.8 (2.8)	8.5 (6.2)	8.1 (4.3)	< 0.001
Suspicious DRE, *n* (%)	569 (21.8)	304 (20.5)	1039 (64.9)	< 0.001
Postoperative upgrading, *n* (%)	183 (7.2)	185 (12.8)	–	< 0.001
Postoperative upstaging, *n* (%)	313 (12.0)	294 (19.8)	627 (39.1)	< 0.001
Pathological tumor stage, *n* (%)				< 0.001
≤ pT2c	2304 (88.4)	1205 (81.2)	999 (62.4)	
≥ pT3a	303 (11.6)	279 (18.8)	603 (37.6)	
Pathological node stage, *n* (%)				< 0.001
pN0	2582 (99.2)	1443 (97.3)	1461 (91.2)	
pN1	21 (0.8)	40 (2.7)	141 (8.8)	
Surgical margin, *n* (%)				< 0.001
R0	1631 (88.8)	1020 (88.4)	1059 (80.4)	
R1	205 (11.2)	134 (11.6)	258 (19.6)	
Pathological Grade Group, *n* (%)				< 0.001
1	1783 (70.6)	543 (37.7)	159 (10.1)	
2/3	86 (3.4)	34 (2.3)	91 (5.8)	
2	512 (20.3)	693 (48.1)	715 (45.5)	
3	96 (3.8)	124 (8.6)	471 (30.0)	
4	49 (1.9)	48 (3.3)	135 (8.6)	
Adjuvant radiotherapy, *n* (%)	89 (3.4)	69 (4.7)	131 (8.2)	< 0.001
Adjuvant hormone therapy, *n* (%)	53 (2.0)	54 (3.6)	124 (7.7)	< 0.001

*PCa* prostate cancer, *FIR* favorable intermediate risk, *UIR* unfavorable intermediate risk, *PSA* prostate-specific antigen, *DRE* digital rectal examination

**Fig. 1:**
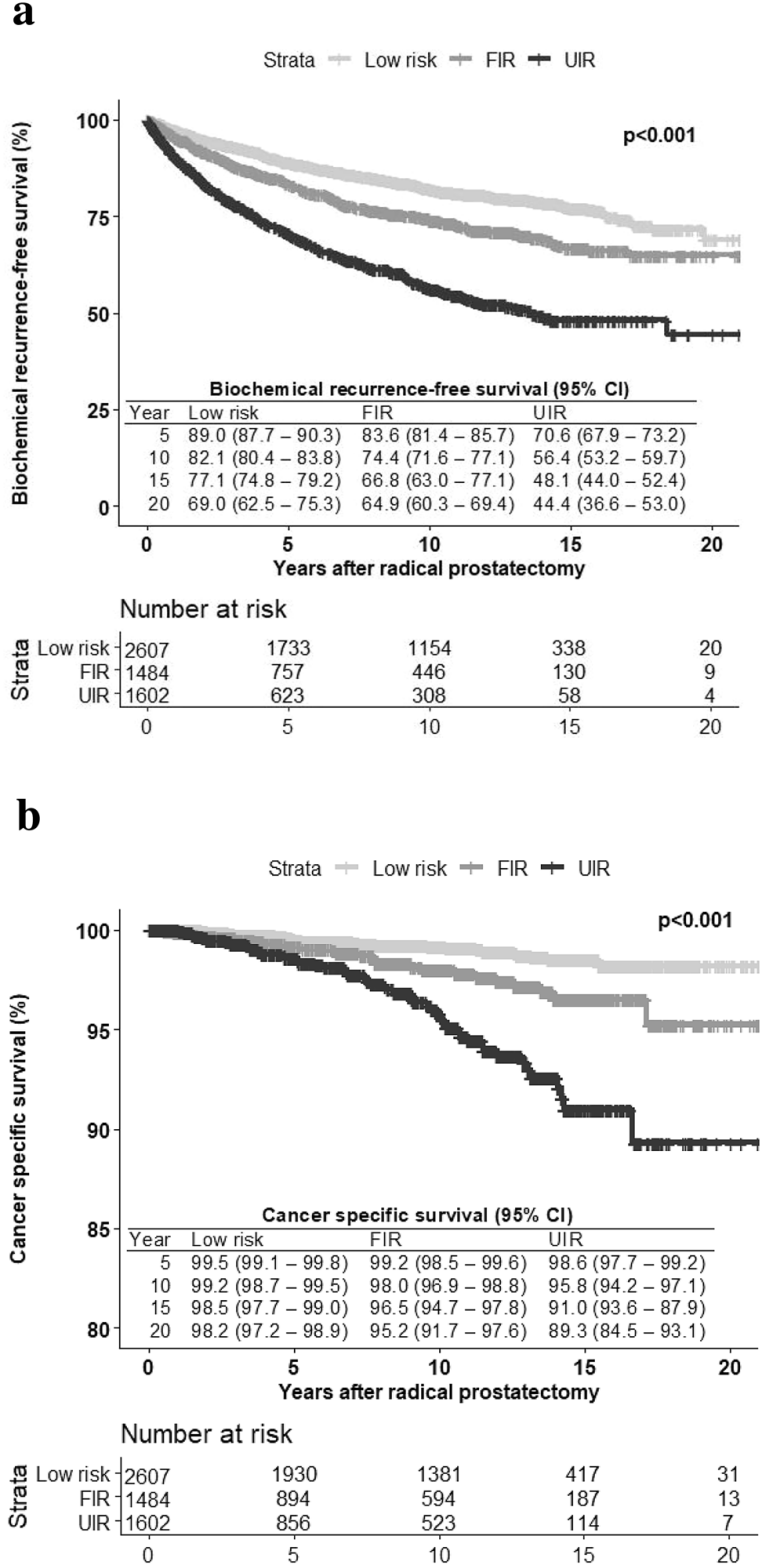
**a** Kaplan–Meier curves for biochemical recurrence-free survival and** b** cancer-specific survival

Kaplan–Meier estimated BRFS rates (Fig. 1a) and CSS rates (Fig. 1b) at 5, 10, 15, and 20 years after RP differed significantly among all three different risk groups (*p* < 0.001). 20-year BRFS was 69%, 65%, and 44% in low-risk, FIR, and UIR PCa patients, respectively (Fig. 1a), while 20-year CSS was 98%, 95%, and 89%, respectively. FIR and UIR PCa were both associated with worse BRFS compared to low risk in the unadjusted Cox regression analysis of risk group alone (HR 1.42, CI 1.17–1.71 for FIR versus low risk; HR 2.83, CI 2.40–3.32 for UIR versus low risk). When adjusting for potential confounder variables, FIR PCa was not associated with worse BRFS compared to low-risk, UIR PCa still was (HR 1.49, CI 1.20–1.85). Concerning CSS, UIR PCa was associated with worse CSS in the single Cox regression (HR 4.69, CI 2.57–8.58), but not after adjustment for potential confounders (Table 2).

**Table 2:** Single and multiple Cox regression of potential prognostic factors for BRFS and CSS

Factors	BRFS						CSS					
	Single Cox regression			Multiple Cox regression^a^			Single Cox regression			Multiple Cox regression^a^		
	HR	95% CI	*p* value	HR	95% CI	*p* value	HR	95% CI	*p* value	HR	95% CI	*p* value
Risk stratification (ref: low-risk PCa)			< 0.001			< 0.001			< 0.001			
FIR PCa	1.42	[1.17; 1.71]		1.07	[0.87; 1.32]		1.62	[0.76; 3.42]				
UIR PCa	2.83	[2.40; 3.32]		1.49	[1.20; 1.85]		4.69	[2.57; 8.58]				
Age at surgery			0.024						0.003			
Continuous	1.01	[1.01; 1.02]					1.07	[1.02; 1.12]				
Family history of PCa (ref: non)			0.532						0.599			
First degree	1.04	[0.87; 1.25]					0.77	[0.38; 1.58]				
Hereditary	1.16	[0.89; 1.52]					1.31	[0.56; 3.07]				
Fatal family history of PCa (ref: non)			0.290						0.976			
Yes	0.81	[0.55; 1.19]					0.98	[0.31; 3.14]				
Other cancer family history (ref: non)			0.783						0.636			
Yes	0.98	[0.85; 1.13]					0.89	[0.53; 1.47]				
Secondary urologic cancer (ref: non)			0.403						0.444			
Yes	0.84	[0.55; 1.28]					0.46	[0.06; 3.34]				
Secondary non-urologic cancer (ref: non)			0.500						0.890			
Yes	0.92	[0.72; 1.18]					0.94	[0.41; 2.19]				
PSA at diagnosis (ng/mL)			< 0.001			< 0.001			< 0.001			0.007
Continuous	1.09	[1.08; 1.11]		1.04	[1.02; 1.06]		1.16	[1.10; 1.22]		1.09	[1.03; 1.17]	
DRE (ref: non-suspicious)			< 0.001						< 0.001			
Suspicious	1.39	[1.21; 1.60]					2.63	[1.57; 4.41]				
Pathological tumor stage (ref: pT2)			< 0.001			< 0.001			< 0.001			0.001
≥ pT3a	3.20	[2.77; 3.69]		1.99	[1.68; 2.36]		7.07	[4.18; 11.96]		2.80	[1.50; 5.22]	
Pathological node stage (ref: pN0)			< 0.001			< 0.001			< 0.001			
pN1	4.74	[3.73; 6.02]		2.04	[1.53; 2.72]		7.80	[3.95; 15.41]				
Surgical margin (ref: R0)			< 0.001			< 0.001			< 0.001			0.024
R1	2.54	[2.16; 2.98]		1.69	[1.39; 2.05]		3.81	[2.25; 6.45]		2.01	[1.09; 3.69]	
Pathological Grade Group (ref: 1)			< 0.001			< 0.001			< 0.001			< 0.001
2/3	2.39	[1.72; 3.31]		1.78	[1.27; 2.49]		3.88	[1.10; 14.34]		2.33	[0.61; 8.97]	
2	1.87	[1.58; 2.23]		1.35	[1.116; 1.635]		4.41	[2.00; 9.72]		3.09	[1.33; 7.16]	
3	3.40	[2.75; 4.19]		1.85	[1.455; 2.360]		7.28	[2.88; 18.45]		3.95	[1.44; 10.84]	
4–5	4.31	[3.35; 5.56]		2.01	[1.499; 2.703]		24.41	[11.03; 54.02]		10.90	[4.37; 27.20]	
Adjuvant radiotherapy (ref: no)			< 0.001			0.004			0.033			
Yes	2.06	[1.62; 2.62]		0.66	[0.500; 0.879]		2.35	[1.07; 5.18]				
Adjuvant hormone therapy (ref: no)			< 0.001						< 0.001			
Yes	2.60	[2.00; 3.36]					5.06	[2.57; 9.99]				

*BRFS* biochemical recurrence-free survival, *CSS* cancer-specific survival, *HR* hazard ratio, CI confidence interval, *PCa* prostate cancer, *FIR* favorable intermediate risk, *UIR* unfavorable intermediate risk, *PSA* prostate-specific antigen

^a^With backward elimination (selection level 5%)

## Discussion

The recognition of the heterogeneity of intermediate-risk PCa has led to increasing interest in expanding the indication for AS to patients with FIR PCa [18]. Although several guidelines have recently added AS as a feasible management option in FIR PCa patients, the safety of this approach remains controversial, since there is no randomized controlled trial comparing this subgroup to low-risk PCa patients and results about long-term outcomes with large sample sizes are lacking. The current study serves as a contemporary review of pathological outcomes and provides long-term outcomes in a large sample of this patient population.

Rates of postoperative upgrading and upstaging were both higher in FIR PCa patients compared to patients at low-risk and those with FIR PCa were more likely to have adverse pathological features including pathological tumor and node stage, pathological GG at RP, and they had more often adjuvant radiotherapy and hormone therapy. The higher rates of postoperative upgrading and upstaging should be considered and discussed with patients if they strongly favor AS, however, it is noteworthy that absolute difference in adverse pathological features such as organ confined disease and pathological node stage was only about 7% and 2%, respectively. Additionally, there was no difference concerning surgical margin. Previous studies showed largely comparable results regarding pathological results [12, 13, 19]. Rates of upgrading and upstaging, respectively, were slightly lower (7.2–19.8% vs. 6.8–27.4%) compared to results in the recent literature [12, 13, 20]. However, it is important to note that definitions varied compared to the current study, since upgrading and upstaging were often combined to the term adverse pathology.

BRFS at 15 years was 77%, 67%, and 48% in patients with low-risk, FIR, and UIR PCa, respectively. Interestingly, absolute difference of long-term BRFS at 20 years became smaller between patients with low risk and FIR [4% (69% vs. 65%)], whereas the difference to UIR was still very large [18% (65% vs. 43%)]. When adjusting for other relevant factors in the multiple Cox regression analysis, FIR PCa was not associated with a worse BRFS (HR 1.07, CI 0.87–1.32). In contrast, UIR PCa was associated with a worse BRFS (HR 1.49, CI 1.20–1.85), which shows that a difference clearly exists between FIR and UIR. This is in line with findings of Aghazadeh et al. investigating a shorter follow-up of 3686 patients after RP. After controlling for year of surgery, FIR did not differ significantly from patients at low risk; however, UIR PCa was associated with a worse 5-year BRFS [12].

In the current study, CSS at 15 and 20 years was high among all three subgroups [99% (low risk) vs. 97% (FIR) vs. 91% (UIR); *p* < 0.001] and [98% (low risk) vs. 95% (FIR) vs. 89% (UIR); *p* < 0.001], respectively. However, CSS rates of FIR were closer to low-risk than to UIR PCa patients. Once again, this emphasizes the existence of FIR as an own risk group and the feasibility of AS in the well-counseled patient. Moreover, after adjusting for other factors neither FIR nor UIR was associated with a worse CSS. In the AS screening arm of the Göteborg trial, CSS at 15 years was lower for the intermediate-risk group compared to our results (90% vs. 97%); however, they did not distinguish between FIR and UIR, explaining the different results [21]. In the Sunnybrook AS cohort, 15-year CSS for intermediate-risk cases was 89%, and for low-risk cases 97%. Whereas the estimates for low-risk cases are comparable to ours, the estimates of intermediate-risk cases are considerably higher. Once again, in the study design, there was no clear differentiation between FIR and UIR cases [9].

The current study provides additional data that a positive family history or a fatal family history of PCa is not associated with a higher risk of worse long-term outcomes, i.e., BRFS and CSS, respectively, in FIR PCa patients. Additionally, recent data from a cross-sectional study of our own group indicated no detrimental effect of a positive family history on postoperative upgrading and upstaging in FIR PCa [22]. Thus, patients at FIR with a positive family history could be reassured that their positive family history is not a reason to refrain from AS if they favor it.

In the current analysis, we observed a significant difference in adverse pathological features between patients at low risk and those at FIR; however, long-term outcomes revealed only slight differences between them. On the other hand, differences between FIR and UIR were very large supporting different treatment strategies between these groups such as AS. However, patients must be informed that the evidence supporting definitive treatment is more robust compared to AS. Additionally, patients should be aware of that when AS is selected as first-line management strategy, a considerable amount of men with FIR eventually proceed with definitive treatment the following years. However, there is evidence that a period of AS does not result in worse outcomes compared to patients undergoing up-front RP [23]. Finally, shared decision-making and adequate patient counseling is paramount in reaching appropriate treatment decisions.

Limitations of the current study include its retrospective nature and that data were derived from a RP cohort so that results may be subject to selection bias. Furthermore, there was not a central pathology review of the biopsy and prostatectomy specimen increasing the risk of inter-observer variation of pathological features. In addition, our database does not provide information about the use of preoperative mpMRI and targeted biopsies in our patient population. This might lead to increased rates of upgrading and upstaging compared to patients diagnosed with mpMRI and targeted biopsies. Therefore, we additionally assessed whether stage and grade migration changed over the study period trying to investigate indirectly the impact of modern diagnostic pathways implemented in the past decade. However, stage and grade migration even increased toward non-organ confined disease/lymph node invasion and higher GG over the study period suggesting that the use of additional mpMRI might be low in the current sample and that there are other unknown factors leading to these increased rates. Furthermore, it should be kept in mind when interpreting results of the current analysis that a patient of a certain risk group in the modern era might differ from one diagnosed one or even two decades ago, particularly in the method of diagnosis. Eventually, data such as perineural invasion, number of positive cores, percentage PCa in a core, and PSA density were lacking and not included in the analysis. Despite these limitations, our data provide important information about long-term outcomes of low-risk, FIR, and UIR PCa patients in a large nationwide, population-based sample with verified, complete, and detailed information about family history of PCa. Nowadays, use of contemporary technology including genomics and mpMRI is steadily increasing and may refine risk stratification, especially in the intermediate-risk group.

## Conclusion

Results of the current study indicated that FIR PCa patients are more likely to have worse pathological outcomes compared to those at low risk. However, the absolute differences are small- and long-term outcomes differed only slightly between low-risk and FIR PCa patients, whereas the difference between FIR and UIR was very large. This emphasizes the role of AS in FIR PCa patients as an appropriate treatment strategy in the well-counseled patient. Nevertheless, shared decision-making and adequate patient counselling have the key role in reaching the best decision for each patient.

## Supplementary Information

Below is the link to the electronic supplementary material.

Supplementary file1 (DOCX 17 KB)

## Data Availability

The datasets generated during the current study are available from the corresponding author on reasonable request.
