# Supplementary material for: Long-term and pathological outcomes of low- and intermediate-risk prostate cancer after radical prostatectomy: implications for active surveillance
Source: World J Urol. 2021 May 10;39(10):3763–70. doi: 10.1007/s00345-021-03717-2 (PMC8521579; doi:10.1007/s00345-021-03717-2)
Supplement: Supplementary file 1 — Supplementary file1 (DOCX 17 KB) [file 345_2021_3717_MOESM1_ESM.docx]

| **Supplementary Table 1** Stage and grade migration depending on year of surgery | | | | |
| --- | --- | --- | --- | --- |
|  | 1994 – 2009 | 2010 – 2014 | 2015 – 2019 | p value |
| Postoperative upgrading^1^ from GG 1 to GG 2, n (%) |  |  |  | <0.001 |
| Yes | 437 (12.8) | 133 (19.6) | 151 (22.1) |  |
| No | 2974 (87.2) | 546 (80.4) | 533 (77.9) |  |
| Postoperative upgrading^1^ from GG 1 to GG ≥3, n (%) |  |  |  | 0.677 |
| Yes | 184 (5.4) | 32 (4.7) | 33 (4.8) |  |
| No | 3227 (94.6) | 647 (95.3) | 651 (95.2) |  |
| Postoperative upgrading^1^ from GG 2 to GG ≥3, n (%) |  |  |  | <0.001 |
| Yes | 136 (4.0) | 65 (9.6) | 93 (13.6) |  |
| No | 3275 (96.0) | 614 (90.4) | 591 (86.4) |  |
| Postoperative upstaging^2^ (pT3-pT4 or pN1), n (%) |  |  |  | 0.021 |
| Yes | 825 (20.9) | 173 (21.7) | 236 (25.0) |  |
| No | 3127 (79.1) | 625 (78.3) | 707 (75.0) |  |
| *GG* Grade Group  ^1^Percentages of postoperative upgrading were calculated based on patients without the presence of Gleason GG 3 on biopsy (n = 4774)  ^2^Percentages of postoperative upstaging were calculated based on all patients (n = 5693) | | | | |
